# Time to treatment failure and its predictors among children receiving first-line antiretroviral therapy in Tigray Region public general hospitals, North Ethiopia, 2024: Retrospective cohort study

**DOI:** 10.1371/journal.pone.0339269

**Published:** 2026-01-12

**Authors:** Ainom Shimondi Shishay, Berhe Beyene Gebrezgiabher, Yohannes Kinfe Gebreyohannes, Birhane Mekonen Negash, Berhe Gebrehiwot Tewele, Efrem Shushay Berhe, Misho Mlaw Kidane, Tetemke Mekonen Tekea, Teklebrhan Kinfe Gebru

**Affiliations:** 1 Department of Public Health, College of Health Science, Adigrat University, Adigrat, Ethiopia; 2 Department of Epidemiology and Biostatistics, School of Public Health, College of Health Science, Aksum University, Aksum, Ethiopia; 3 School of Medicine, College of Health Science, Aksum University, Aksum, Ethiopia; 4 Araya Kahsu College of Health Science, Aksum, Ethiopia; University of the Witwatersrand, SOUTH AFRICA

## Abstract

**Background:**

Pediatric human immunodeficiency virus (HIV) remains a significant public health challenge, with an estimated 1.5 million children living with HIV globally. In addition, first-line antiretroviral therapy (ART) treatment failure has remained high, as studies and reports showed. Furthermore, time to treatment failure and its predictors on first-line ART among HIV-infected children are less researched in the study area after the test-and-treat strategy is implemented. Hence, this study was conducted to assess time to treatment failure and its predictors among children receiving first-line antiretroviral therapy in Tigray Region public general hospitals, North Ethiopia, 2024.

**Methods:**

A hospital-based retrospective cohort study was conducted among children who started ART from January 2014 to March 2020 and from February 2023 to August 2023 in Tigray Region public general hospitals. Epi Data version 3.1 and Stata version 14 were used for data entry and analysis, respectively. Kaplan-Meier and log-rank tests were computed. Bivariable analysis variables with p-value < 0.2 were taken to multivariable Cox regression analysis to identify predictors. Finally, 95% CI and p-value <0.05 were considered for statistical significance.

**Results:**

From 410 records of children, 55 (13.4%) (95% CI, 10.43–17.08) had treatment failure, with an incidence rate of 3.3 (95% CI, 2.6–4.3) per 1000 child-month observation. The median time to treatment failure was greater than or equal to 75 months. Poor adherence (AHR = 3.6, 95% CI: 1.7–7.5), baseline CD4 count <200 cells/mm^3^ (AHR = 3.8, 95% CI: 1.8–8.4), baseline CD4 count 201–350 cells/mm^3^ (AHR = 2.7, 95% CI: 1.2–6.2), and initial Nevirapine-based regimen (AHR = 4, 95% CI: 1.8–8.8) were predictors of time to treatment failure.

**Conclusion and recommendation:**

The incidence of treatment failure among children receiving first-line ART was found to be high according to the UNAIDS virological suppression targets. Poor adherence, baseline CD4 count, and initial NVP-based regimen were predictors of time to treatment failure of ART. Hence, all children on ART should be closely monitored, mainly on these identified predictors.

## Introduction

Human immunodeficiency virus (HIV) causes acquired immune deficiency syndrome (AIDS). It weakens the immune system and makes the body vulnerable to secondary and opportunistic infections [1]. HIV/AIDS has been recognized as one of the most devastating epidemics ever witnessed in the world since its recognition in 1981, and its impact extends beyond public health concerns [2]. Individuals with HIV/AIDS use antiretroviral therapy (ART) to prolong their life expectancy. ART was started in 1987 and decreased the rate of death from HIV/AIDS, but globally, ART treatment failure has become a common problem [3]. ART treatment failure refers to a suboptimal response or a lack of sustained response to therapy, excluding cases of immune reconstitution inflammatory syndrome [4]. It can be determined through the examination of clinical failure (clinical criteria), immunologic failure (CD4 criteria), virologic failure (viral load criteria), or a combination of all. Viral load is the recommended monitoring approach to confirm treatment failure; in settings where viral load is not routinely available, CD4 count and clinical monitoring should be used to diagnose treatment failure [4,5].

Pediatric HIV remains a significant public health challenge, with an estimated 1.5 million children living with HIV/AIDS worldwide as of 2023 [2]. Similarly, in that report, of the 1.5 million children living with HIV, 91% have started antiretroviral therapy. Moreover, of those on ART follow-up, 81% had only experienced viral suppression [2]. Based on the joint United Nations Programs on HIV/AIDS (UNAIDS) 2023 report, 630,000 AIDS-related deaths occurred globally. Of these AIDS-related deaths, 385,300 deaths occurred in Africa, which is the most affected and hardest-hit region [2]. In Ethiopia, of the children who were taking ART, 21.1% did not experience viral suppression [6]. Furthermore, in 2021, based on the Ethiopian Public Health Institute, 1892 and 181 children living with HIV/AIDS died annually in Ethiopia and Tigray, respectively [7]. In addition, for those infants and children who developed treatment failure of first-line ART, recommending potent and effective second-line regimens is difficult because treatment options are largely nonexistent in most low-income countries [8,9]. Consequently, these challenges emphasize the significance of choosing potent first-line regimens and the necessity to make maximal efforts to ensure increased durability of first-line regimens [9].

Available evidence revealed that treatment failure of ART among children is affected by baseline WHO stage [10–12], adherence [10,12–14], a low CD4 count [11,15], baseline opportunistic infection [16], baseline tuberculosis, hemoglobin [12], caregiver being widowed [15], being malnourished [17], missing cotrimoxazole preventive therapy [10], being orphaned [12,14], and having a nevirapine-based regimen [13,18,19]. But these above-listed predictors were identified either before the test and wait strategy period or during the combined period (including both children who started ART before and after test- and-treat strategies). That is why this study was conducted to ensure whether the predictors of first-line ART remained the same after test-and-treat guidelines were implemented since the level of treatment failure has remained high, as studies and reports indicated.

In addition, UNAIDS and the National Health Sector Transformation Plan (HSTP) outline targets to end the HIV epidemic by ensuring that 95% of those diagnosed with HIV receive sustained ART and 95% of those on ART achieve viral suppression [2,5]. However, treatment failure in first-line ART has remained high [2,6,20]. Besides this, time to treatment failure and its predictors among children receiving first-line ART after implementing test-and-treat guidelines are less researched in Ethiopia; particularly, we did not find any study conducted in the Tigray Regional State after the test-and-treat strategy was implemented. Moreover, even if some studies and reports determined the magnitude of treatment failure, the median time to treatment failure was not well documented after the era of the test-and-treat strategy. Therefore, this study aimed to assess time to treatment failure and its predictors among children receiving first-line antiretroviral therapy from the era of test-and-treat guidelines applied.

## Methods

### Study area and setting

The study was carried out in public general hospitals of the Tigray Regional State. Tigray is one of the twelve regions of Ethiopia and is found in the northern part of Ethiopia. Its capital city is Mekelle, which is located 783 km away from Addis Ababa, the capital city of Ethiopia. According to the Central Statistics Agency of Ethiopia (CSA) 2007 housing and population census projections, the population in the region is estimated to be 5,541,736 with a sex composition of 49% male and the remaining female in 2017. [21]. In 2019, according to the Tigray Region Health Bureau, there were two specialized referral hospitals, 14 general hospitals, 22 primary hospitals, 202 health centers, and 712 health posts. Based on the Ethiopian Public Health Institute’s HIV and AIDS estimations for 2022, in the Tigray Region, a total of 3,869 children (aged 0–14) lived with HIV, and 155 children (aged 0–14) were newly infected [7]. In addition to other services, all general hospitals in the Tigray Regional State provide chronic HIV care (ART) services.

### Study design and period

A hospital-based retrospective cohort study was conducted from March 1–30, 2024.

### Source of population

The source of the population was HIV/AIDS-infected children below 15 years who were taking first-line ART in public general hospitals in the Tigray Region, northern Ethiopia.

### Study population

All HIV/AIDS-infected children under 15 years on first-line ART for at least six months in the selected public general hospitals of the Tigray Region and those who initiated ART from January 1, 2014, up to March 31, 2020, and from February 1, 2023, to August 31, 2023.

### Inclusion and exclusion criteria

#### Inclusion criteria.

All HIV/AIDS-infected children who were below 15 years old, taking first-line ART for at least six months, and who initiated ART from January 1, 2014, up to March 31, 2020, and from February 1, 2023, to August 31, 2023, were included in this study.

#### Exclusion criteria.

Children who had none of the three treatment failure measuring criteria (CD4 count, virological load, or clinical stage) available in their medical records.

### Sample size determination

Sample size was calculated through the Cox model with the assumption of the Cox proportional hazard model by using STATA version 14 software with the assumption of 95% CI, 80% power, 0.5 standard deviation, and by taking the hazard ratio of tuberculosis at baseline 2.27 and the probability of treatment failure 0.23 [20]. Then, multiplied by two (2) for the sake of the design effect, the multistage sampling technique was applied, and finally, a 10% non-response rate was added to account for lost medical charts or incomplete information (if >10% of the variables were missed in medical cards). The total sample size needed for the study was 434, with an expected number of events of 47.

### Sampling procedure and technique

**Fig 1:**
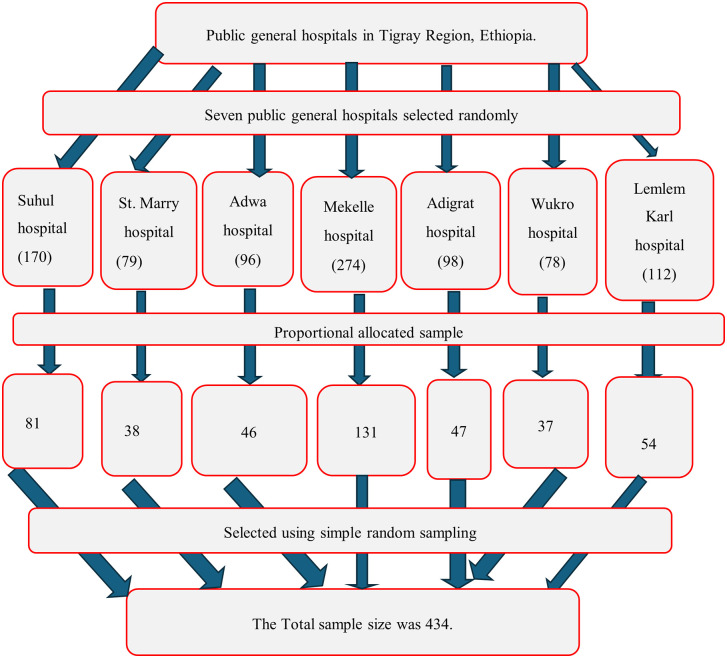
Schematic presentation of sampling procedure to assess time to treatment failure and its predictors among children receiving first-line antiretroviral.

A multistage sampling method was used to select the study participants. Of the fourteen public general hospitals in the Tigray Regional State, seven public general hospitals were selected randomly. The selected public general hospitals were Suhul, Sanit Marry, Adwa, Adigrat, Wukro, Mekelle, and Lemlem Karl public general hospitals. Then, to select the study participants from each selected hospital, the sample size was proportionally allocated. After that, the medical records of children who started first-line ART from January 1, 2014, up to March 31, 2020, and from February 1, 2023, to August 31, 2023, were retrieved from the ART register. Using the ART register, simple random sampling through a computer-generated method was used to select the study participants from the medical records (Fig 1). The period from April 1, 2020, up to January 30, 2023, was excluded due to the war, which collapsed the health care system in the Tigray Region. Hence, during that time there were no health services that led to inadequate data or incomplete records. That is why this period was excluded from the study period.

### Operational definitions

**Time to treatment failure:** The time between ART initiation and detection of treatment failure of first-line ART [13]. The time to treatment failure is measured in months.

**Event (1):** Was HIV/AIDS-infected children below 15 years who experienced treatment failure based on WHO criteria, which are either clinical, immunological, and/or virological failure after six months of effective ART treatment such that at least one criterion should be fulfilled [5,22].

**Children:** individuals who are less than 15 years old [5].

**Clinical failure:** New or repeated clinical events indicating advanced or severe immune deficiency (WHO clinical stage 3 and 4 clinical conditions except for TB) after 6 months of effective treatment [5].

**Immunological failure:** Persistent (at least 2 CD4 measurements) CD4 levels below 200 cells/mm for children younger than 5 years and CD4 levels below 100 cells/mm for older than 5 years [5].

**Virological failure:** when viral load is above 1000 copies/mL on two consecutive viral load measurements (i.e., measuring after 3 months from the first measurement), with adherence support for 3 months following the first viral load test [5].

**Censored (0):** Those who did not experience treatment failure during follow-up time, including defaulters, transfer outs, died, or exceeded 15 years of age during follow-up. The time to occurrence of an event or censored cases was measured in months [20].

**Adherence:** The extent to which a client’s behavior coincides with the prescribed regimen as agreed upon through a shared decision-making process between the client and the health care provider (13), evidenced from follow-up cards. A combination of tools was used to assess patient medication adherence. Based on the remaining pill count, clinicians can consider good, fair, and poor adherence. We consider good, fair, and poor adherence if the percentage of adherence dose is ≥ 95%, 85–94%, and <85%, respectively [16].

**WHO clinical stage:** In this study the WHO clinical stage was categorized as early stage (WHO stage I and II) and advanced stage (WHO stage III and IV) [4,5].

**First-line ART regimen/therapy:** In Ethiopia, the first-line ART regimens that existed during this study duration (2014–2023) were, for children younger than 3 years old, the first-line ART is ABC or AZT + 3TC + LPV/r, or as an alternative, ABC or AZT + 3TC + NVP. For children 3 years to less than 10 years and adolescents *<*30 kg, the regimen AZT/ABC + 3TC + EFV is the preferred first-line, while ABC/AZT + 3TC + NVP or TDF + 3TC + EFV/NVP are alternative first-line regimens, and adolescents (10–19 years) ≥30 kg receive TDF + 3TC + EFV (FDC)/DTG as first-line ART therapy or, as an alternative, TDF + 3TC + NVP/EFV, AZT + 3TC + NVP, ABC/AZT + 3TC + EFV, and AZT + 3TC + DTG/NVP. In addition, for children > 4 weeks and > 3 kg but less than 10 years old, the first-line ART regimen is ABC + 3TC + DTG, or as an alternative first-line ART regimen, ABC + 3TC + LPV/r or AZT + 3TC + DTG [5,23,24].

### Data collection instruments and procedure

A structured data extraction checklist was developed from the national comprehensive HIV treatment guideline, the ART registration booklet, the ART monitoring multi-chart, and a review of previous related studies. The checklist comprises socio-demographic characteristics, clinical and laboratory characteristics, ART follow-up, and other medication characteristics. The lists of participants were taken from the ART data clerk and medical records number, or unique ART numbers, which helped to find charts in the hospital card room. The time to the occurrence of treatment failure was measured in months. Five data collectors of BSc nurses and two supervisors working in an ART clinic were recruited, and the data collection was conducted from March 1–30, 2024.

### Data processing and analysis

The collected data was entered into Epi-data version 3.1, then exported, cleaned, edited, coded, and analyzed using Stata version 14. Exploratory analysis was carried out to see missing values and influential outliers. The WHO Anthro Plus software was used to classify index variables or assess the nutritional status of the child. Then the data was described with relative frequency, percent, tables, graphs, and charts, and the outcome of each subject was dichotomized into failure and censored. Time to treatment failure of patients was obtained from medical cards by calculating the time between the date of ART initiation and the occurrence of an event or censoring. The incidence density rate (IDR) was calculated for the entire study period. The Kaplan-Meier survival curve was used to estimate the median time to treatment failure and the cumulative probability of treatment failure. A log-rank test was used to compare failure curves between different categories of explanatory variables. A life table was used to estimate the probabilities of failure at different time intervals. Assumptions for the Cox proportional hazard model were checked using the Schoenfeld residuals test (global test P-value = 0.64) and graphically with the log-log Cox adjusted survival estimate (parallel plot of survival). The model’s fitness was checked using the Nelson-Aalen cumulative hazard rate relative to Cox-Snell residuals (the line lay between 0 and 1, and it was 45 degrees). Multicollinearity was checked, and no independent variable was found to have multicollinearity using tolerance and variance inflation factor (mean VIF = 1.45). Bivariable analysis was done to identify predictors associated with dependent and independent variables with a p-value of <0.2. Then these variables were entered into multivariable analysis to identify predictors associated with treatment failure with a p-value of <0.05 through the Cox proportional hazard regression model. The hazard ratio (HR) with a 95% confidence interval was computed, and statistical significance was declared at the 5% level (p-value < 0.05).

The model for Cox proportional hazard is [25].

hx(t) = h0(t)exp[βixi]

where hx(t) = hazard function, h0(t) = baseline hazard, and exp[βixi] = function reflects how the hazard function changes according to differences in subjects’ characteristics.

### Data quality assurance

Data quality was assured by the proper data extraction format and through continuous supervision. Two supervisors and the principal investigator closely supervised the entire data collection process. Moreover, the data collectors were working outside of the selected study hospitals. One-day training about the objectives, significance, and variables of the research and how to extract the data using the data extraction format was given to the data collectors and supervisors. The checklist was pretested on 5% (22) of randomly selected charts in Axum Saint Marry public general hospital prior to one week of the actual study, and then its clarity, wording, and logical sequence of the format were ensured. Moreover, these 22 charts were not included in the study. To assure the completeness of the collected data, all the collected data was checked at the end of each day by the principal investigator and supervisors. Whenever there appears to be incompleteness or uncertainty in the recording, the filled-in information format is cross-checked with the source data. The collected data was checked for completeness prior to data entry. Finally, data exploration on the entered data was made to see unexpected values and outliers.

### Ethical consideration

The study was carried out after gaining ethical approval from the institutional review board (IRB) of Aksum University College of Health Sciences and Specialized Referral Hospital, with a reference (IRB Number: 14/2024). After being approved by the IRB and getting permission from the Regional Health Bureau (RHB), an official letter of cooperation was written from the RHB for all selected hospitals with a reference number (Ref 1342/7767/16). At each selected hospital, after handing over a letter of cooperation, permission was received from each selected hospital on behalf of patients since the study was conducted through secondary data of reviewing softcopy databases and medical cards. The recruited data collectors and supervisors had worked at an ART clinic and ART data clerks and were well trained in handling patients’ data securely and in maintaining confidentiality.

Through these above procedures, the required data was retrieved anonymously from the soft copy database of the ART data clerk and/or by reviewing medical records. Greatest patient confidentiality was taken through the whole process. Given the retrospective nature of the study involving soft copy database and/or medical record review, the risk of harm to individual patients is minimal as long as confidentiality is strictly maintained. Moreover, to guarantee confidentiality, the collected data was anonymized using codes, stored securely in a locked box, and entered into a password-protected computer. Patient names were not included in the data collection format. The principal investigator remained the only individual with access to the data. All methods were performed in accordance with the Declaration of Helsinki.

## Results

### Socio-demographic characteristics

A total of 434 medical cards of children who started ART drugs were reviewed, of which 24 (5.5%) were excluded due to the incompleteness of medical records. Finally, 410 (94.5%) medical records were left in the final analysis. More than half of the medical records of children had missing values in the baseline viral load variable at ART initiation. Therefore, the baseline viral load variable is handled by the stepwise deletion method. Except for religion, which has 4 (1%) missing values, and marital status of the caregiver, which has 3 (0.7%) missing values, all variables have complete data. Hence both variables have <2% missing values, considered as they have no effect on the result and are handled by the imputation method of mode. Almost half (49.3%) of the study participants were female. The mean age of the study participants at ART enrollment was 6.7 with SD ± 3.94. Almost one-third (33.9%) of the children were under 5 years old. Most study participants (80.7%) were living in urban areas (Table 1).

**Table 1:** Socio-demographic characteristics of children receiving first-line antiretroviral therapy in Tigray region public general hospitals, north Ethiopia, from January 1, 2014, up to March 31, 2020, and from February 1, 2023, to August 31, 2023 (N = 410).

Independent variables	Category	Status		
		Failure N (%)	Censored N (%)	Total N (%)
Sex	Male	32(15.4)	176(84.6%)	208(50.7%)
	Female	23(11.4%)	179(88.6%)	202(49.3%)
Age at ART initiation	<5	20(14.4%)	119(85.6%)	139(33.9%)
	5-9	21(14.4%)	125(85.6%)	146(35.6%)
	10-15	14(11.2%)	111(88.8%)	125(30.5%)
Residency	Urban	44(13.3%)	287(87.7%)	331(80.7%)
	Rural	11(13.9%)	68(86.1%)	79(19.3%)
Marital status	Single	3(10.7%)	25(89.3%)	28(6.8%)
	Married	34(11.8%)	253(88.2%)	287(70%)
	Divorced	6(13.3%)	39(86.7%)	45(11%)
	Widowed	12 (24%)	38(76%)	50(12.2%)
Level of education	No education	33(15.4%)	182(84.6%)	215(52.4%)
	Primary	8(10%)	72(90%)	80(19.5%)
	Secondary	7(15.9%)	37(84.1%)	44(10.7%)
	Tertiary	7(9.9%)	64(9.1%)	71(17.3%)
Occupation	Farmer	12(19.1)	51(80.9%)	63(15.4%)
	Marchant	11(9.4%)	106(90.6%)	117(28.5%)
	Governmental employee	10(12.2%)	72(87.8%)	82(20%)
	Daily labor	10(14.7%)	58(85.3%)	68(16.6%)
	Housewife	10(15.9%)	53(84.1%)	63(15.4%)
	Others*	2(11.8%)	15(88.2%)	17(4.1%)
Religion	Orthodox	44(13.2%)	290(86.8%)	334(81.5%)
	Muslim	10(16.4%)	51(83.6%)	61(14.9%)
	Others **	1(6.7%)	14(93.3%)	15(3.7%)
primary caretaker	Both parents	25(11.7%)	189(88.3%)	214(52.2%)
	Mother	22(16.4%)	112(83.6%)	134(32.7%)
	Father	6(15.8%)	32(84.2%)	38(9.3%)
	Relatives	2(9.5%)	19(90.5%)	21(5.1%)
	Orphanage	0(0%)	3(100%)	3(0.7%)
Caretaker serology status	Negative	2(7.7%)	24(92.3%)	26(6.3%)
	Positive	38(12.5%)	265(87.5%)	303(73.9%)
	Unknown	15(18.5%)	66(81.5%)	81(19.8%)

*Other occupations: tailor and nongovernment employee.

**Other religions: Catholic and Protestant.

### Baseline clinical, immunological, and nutritional characteristics

Of the 410 children receiving ART, 165 (40.2%) had advanced baseline WHO stages (stages III and IV) during the initiation of ART. Regarding the baseline CD4 count of study participants, 44 (10.7%) had < 200 cells/mm³, 59 (14.5%) had 200–350 cells/mm³, 63 (15.4%) had 351–500 cells/mm³, and 244 (59.9%) had > 500 cells/mm³. About The nutritional status of children at baseline showed that 72 (17.6%) were wasted and 79 (19.2%) were stunted. Moreover, on the status of malnutrition severity, 41 (10%) were severely wasted and 33 (8%) were severely stunted (Table 2).

**Table 2:** Baseline clinical, immunological, and nutritional characteristics of children receiving first-line antiretroviral therapy in Tigray region public general hospitals, north Ethiopia, from January 1, 2014, up to March 31, 2020, and from February 1, 2023, to August 31, 2023 (N = 410).

Independent variables	Category	Status		
		Failure N (%)	Censored N (%)	Total N (%)
Opportunistic infection	No	7(3.1%)	220 (96.9%)	227 (55.4%)
	Yes	48 (26.2%)	135 (73.8%)	183 (44.6%)
Tuberculosis	No	50(12.9)	336(87.1%)	386(94.1%)
	Yes	5(20.8%)	19(79.2%)	24(5.9%)
WHO clinical stage	Early stage	8(3.3%)	237(96.7%)	245(59.8%)
	Advanced stage	47(28.5%)	118(71.5%)	165(40.2%)
CD4 count (cells/mm^3^)	<200	18(40.9%)	26(59.1%)	44(10.7%)
	200−35	14(23.7%)	45(76.3%)	59(14.4%)
	351-500	8(12.7%)	55(87.3%)	63(15.4%)
	>500	15(6.2%)	229(93.8%)	244(59.9%)
Hemoglobin	Not anemic	35(11.8%)	262(88.2%)	297(72.4%)
	Anemic	20(17.7%)	93(82.3%)	113(27.6%)
Developmental history for age < 5 years	Appropriate	14(13.7%)	88(86.3%)	102(24.9%)
	Delayed	3(14.3%)	18(85.7%)	21(5.1%)
	Regression	3(20%)	12(80%)	15(3.7%)
Functional status for age > 5 years	Working	5(10%)	45(90%)	50(18.4%)
	Ambulatory	28(13.2)	184(86.8%)	212(77.9%)
	Bedridden	2(20%)	8(80%)	10(3.7%)
BAZ (Body Mass Index-for- Age) (wasting/thinness)	Normal	43(12.7%)	295(87.3%)	338(82.4%)
	Moderate wasted	5(16.1%)	26(83.9%)	31(7.6%)
	Sever wasted	7(17.1%)	34(82.9%)	41(10%)
Height For age (HFA) (stunting)	Normal	48(14.5%)	283(85.5%)	331(80.7%)
	Moderate	2(4.4%)	44(95.6%)	46(11.2%)
	Severe	5(15.2%)	28(84.8%)	33(8%)

CD4= Clusters of Differentiation-4 cells, WHO= World Health Organization.

### ART and follow-up characteristics of children on ART

Of the total 410 children on ART, 60 (14.6%) and 39 (9.5%) had poor and fair adherence, respectively. Among the study participants, 140 (34.1%) had an NVP-based regimen at the time of starting ART. Moreover, of these study participants, 244 (59.5%) took isoniazid prophylaxis, and 282 (68.8%) took cotrimoxazole prophylaxis. From the total number of children who initiated ART during the study period, 10 (2.44%) of them died, 339 (82.7%) were on ART, 7 (1.7%) lost follow-up, and 54 (13.2%) transferred out. Regarding the substitution or change of ART regimen, of the 410 study participants, 125 (30.5%) changed their ART regimen. Of these substituted or changed ART regimens, 83 (66.4%) were due to the new ART regimen, 24 (19.2%) had treatment failure, 5 (4%) had TB infection, 3 (2.4%) had toxicity, and 10 (8%) others (Table 3).

**Table 3:** ART and follow-up characteristics of children receiving first-line antiretroviral therapy in Tigray region public general hospitals, north Ethiopia, from January 1, 2014, up to March 31, 2020, and from February 1, 2023, to August 31, 2023 (N = 410).

Independent variable	Category	Status		
		Failure N (%)	Censored N (%)	Total N (%)
Initial ART regimen	Non NVP based	8(3%)	262(97%)	270(65.9%)
	NVP based	47(33.6%)	93(66.4%)	140(34.1%)
ART adherence	Good	28(9%)	283(91%)	311(75.9%)
	Fair	7(18%)	32(82%)	39(9.5%)
	Poor	20(33.3%)	40(66.7%)	60(14.6%)
Disclosure status	Yes	13(10.6%)	110(89.4%)	123(30%)
	No	42(14.6%)	245(85.4%)	287(70%)
PMTCT exposure	Given	15(14.4%)	89(85.6%)	104(25.4%)
	Not given	40(13.1%)	266(86.9%)	306(74.6%)
Isoniazid prophylaxis	Given	32(13.1%)	212(86.9%)	244(59.5%)
	Not given	23(13.9%)	143(86.1%)	166(40.5%)
CPT prophylaxis	Given	39(13.8%)	243(86.2%)	282(68.8%)
	Not given	16(12.5%)	112(87.5%)	128(31.2%)
Regimen change	Yes	24(19.2%)	101(80.8%)	125(30.5%)
	No	31(10.9%)	254(89.1%)	285(69.5%)
ART side effect	Yes	6(18.2%)	27(81.8%)	33(8%)
	No	49(13%)	328(87%)	377(92%)

PMTCT = Prevention of Mother-To-Child Transmission.

CPT = Cotrimoxazole Preventive Therapy.

In addition, about the types of ART side effects, of the 410 study participants of reviewed charts, 33(8%) developed ART side effects. Out of these developed ART side effects were 6 (18.18%) nausea, 6 (18.18%) fatigue, 9 (27.27%) headache, 6 (17.65%) skin rash, 3 (9.09%) anemia, and 3(9.09%) others.

### Time to treatment failure and its incidence after ART initiation

**Fig 2:**
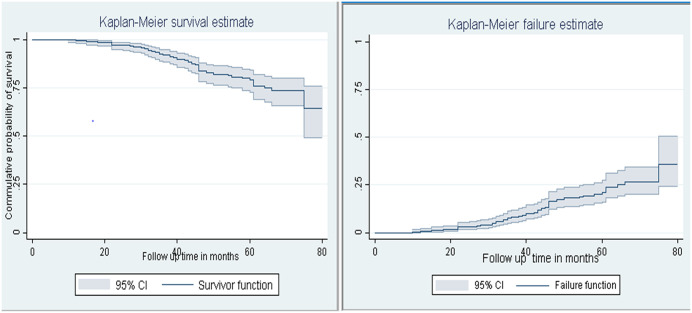
The overall Kaplan-Meier survival and failure estimate of children receiving first-line antiretroviral therapy.

After initiation of ART, children were followed for different periods of time: a minimum of 7 months and a maximum of 80 months, with a median follow-up time of 38 months and an IQR of 29 (24–57). The median time to treatment failure was undetermined, but its 95% CI of the lower limit was determined to be 75 months (95% CI, 75 - ___) (Fig 2). But, when estimated, the survival time at which the cumulative survival function is equal to 0.5 by the restricted mean was 70.1 months; this mean was underestimated since the largest observed time was censored; in addition, when estimated by the extended mean, it was 186.8 months, which was too long compared to the determined 75 months of the 95% CI of the lower limit. Hence, the 95% CI lower limit was determined to be 75 months; therefore, the median time to treatment failure was greater than or equal to 75 months.

The children were followed for 16,532 child-month observations, which makes the incidence rate of treatment failure 3.3 (95% CI, 2.6–4.3) per 1000 child-months of observation or 39.6 per 1000 child-years of observation. Moreover, of the 410 study participants, 13.4% (95% CI, 10.43–17.08) of the children developed first-line ART treatment failure. Of these 55 (13.4%) treatment failures, 31 (56.4%) had virological failure, 7 (12.7%) had immunological failure, 5 (9.1%) had clinical failure, 8 (14.5%) had immunological and virological failure, 3 (5.4%) had immunological and clinical failure, and 1 (1.8%) had virological and clinical failure.

### Overall failure functions

The overall Kaplan-Meier estimate showed that the probability of developing treatment failure among children receiving first-line ART was low in the first month of ART initiation and relatively increased as follow-up time increased. The cumulative probabilities of developing treatment failure were 35.7% at the end of the study. Moreover, cumulative probabilities of developing treatment failure at the end of 12, 24, 36, 48, 60, and 72 months of follow-up were 0.25% (95% CI, 0.04–1.78), 2.79% (95% CI, 1.51–5.13), 7.39% (95% CI, 4.96–10.96), 16.01% (95% CI, 11.94–21.28), 20.17% (95% CI, 15.31–26.32), and 28.16% (95% CI, 21.18–36.83), respectively (Fig 2).

### Assessment of model adequacy, goodness of fit, and Cox proportional hazard assumptions

#### Overall assessment of model adequacy.

The value of Harrell’s C was 0.86, which indicates that we can correctly order survival times for pairs of patients 86% of the time on the basis of measurement-fitted variables in the model. The C index is defined as the proportion of all usable subject pairs in which the predictions and outcomes are concordant.

#### Checking residuals.

Residuals are checked by the Cox-Snell residual plot. The plot showed that the hazard follows the 45-degree line very closely, we conclude that the data fit well (S1 Appendix I)

#### Proportional hazard model assumption.

The proportional hazard model assumption is one of the most important assumptions in the Cox model. Graphical and statistical methods were used to assess the assumption. The Cox proportional hazard model assumption was checked by using the Schoenfeld residual test (global test). The proportional hazard assumption is rejected if the p-value is < 0.05. We observed that each covariate (p-value > 0.05) and all the covariates simultaneously (global for Cox proportional hazard p-value = 0.65 > 0.05) met the proportional hazard assumption (S2 Appendix II).

### Predictors of time to treatment failure

Both bivariable and multivariable analyses were done to assess the association with the occurrence of time to treatment failure among children on ART using the Cox proportional hazard regression model. In the multivariate analysis, baseline CD4 levels below 200 cells/mm^3^ and 200–350 cells/mm^3^, poor adherence, and NVP-based regimens remained statistically significant predictors of treatment failure among children on ART.

According to our analysis, the hazard of treatment failure was 3.8 times higher among children with a CD4 count less than 200 cells/mm³ than those with a CD4 count above 500 cells/mm³ (AHR = 3.8, 95% CI: 1.8–8.5). Moreover, these children with a CD4 count of 200–350 cells/mm³ were 2.7 times more at high risk of developing treatment failure compared to those with a CD4 count above 500 cells/mm³ (AHR = 2.7, 95% CI: 1.2–6.2). The children who initiated the NVP-based regimen were 4 times more at high risk of treatment failure than children who initiated the non-NVP-based regimen (AHR = 4, 95% CI: 1.8–8.8). Regarding adherence to ART drugs, the risk of treatment failure among children who had poor adherence was 3.6 times higher compared to those who had good adherence (AHR = 3.6, 95% CI: 1.7–7.5) (Table 4).

**Table 4:** Cox proportional hazard regression analysis for the predictors of time to treatment failure among children receiving first-line antiretroviral therapy in Tigray region public general hospitals, north Ethiopia, from January 1, 2014, up to March 31, 2020, and from February 1, 2023, to August 31, 2023 (N = 410).

Covariates	Category	Status		CHR (95%CI)	AHR (95%CI)
		Failure N (%)	Censored N (%)		
Sex	Male	32(58.2%)	176(49.6%)	1	1
	Female	23(41.8%)	179(50.4%)	0.69(0.4-1.2)	0.74(0.4-1.4)
Marital status	Married	34(61.8%)	253(71.3%)	1	1
	Single	3(5.5%)	25(7%)	0.85(0.3-2.7)	2.3(0.4-14)
	Divorced	6(10.6%)	39(11%)	1.1(0.5-2.6)	0.87(0.4-2.2)
	Widowed	12 (21.8%)	38(10.7%)	2.1(1.1-3.9) ^*^	1.8(0.8-4.2)
Caregiver Serology Status	Positive	38(69.1%)	265(74.6%)	1	1
	Negative	2(3.6%)	24(6.8%)	0.63(0.2-2.6)	0.17(0.0-1.6)
	Unknown	15(27.3%)	66(18.6%)	1.7(0.9-3.1)	1.4(0.7-2.9)
Opportunistic Infection at baseline	No	7(12.7%)	220 (62%)	1	1
	Yes	48 (87.3%)	135 (38%)	7.6(3.5-17.0) ^**^	2(0.5-8.7)
Baseline WHO clinical stage	Early stage	237(66.8%)	8(14.5%)	1	1
	Advanced stage	118(33.2%)	47(85.5%)	7.8(3.7-16.6) **	2(0.5-8.4)
Baseline CD4 count	<200 cells/mm^3^	18(32.7%)	26(7.3%)	6.5(3.3-13) ^**^	3.8(1.8-8.4) **
	200-35cells/mm^3^	14(25.5%)	45(12.7%)	4.3(2.0-9.1) **	2.7(1.2-6.2) *
	351-500 cells/mm^3^	8(14.5%	55(15.5%)	2.3(0.9-5.47)	1.2(0.5-3.2)
	>500 cells/mm^3^	15(27.3%)	229(64.5%)	1	1
Baseline hemoglobin level	Not anemic	35(63.6%)	262	1	1
	Anemic	20(36.4%)	93(26.2%)	1.7(1.0-3.07) *	1.2(0.6-2.5)
Initial ART regimen	Non NVP based	8(14.5%)	262(73.8%)	1	1
	NVP based	47(85.5%)	93(26.2%)	6.9(3.2-14.7) **	3.9(1.8-8.8) **
Adherence	Good	28(50.9%)	283(79.7%)	1	1
	Fair	7(12.7%)	32(9%)	1.74(0.8-4)	0.8(0.3-2.4)
	Poor	20(36.4%)	40(11.3%)	4.2(2.3-7.5) **	3.6(1.7-7.5) **
ART side effect	Yes	6(10.9%)	27(7.6%)	1.3(0.6-3.1)	1.5(0.6-3.8)
	No	49(89.1%)	328(92.4%)	1	1
BAZ (wasting/thinnest)	Normal	43(78.2%)	295(83.1%)	1	1
	Moderate wasted	5(9.1%)	26(7.3%)	1.19(0.5-3.0)	1.1(0.4-3.4)
	Sever wasted	7(12.7%)	34(9.6%)	1.7(0.8-3.8)	2.1(0.8-5.4)
HFA (Stunting)	Normal	47(85.5%)	314(88.5%)	1	1
	Moderate	4(7.3%)	27(7.6%)	0.32(0.1-1.3)	0.5(0.1-2.1)
	Severe	4(7.3%)	14(3.6%)	1.4(0.6-3.5)	1.9(0.6-5.5)

NB: * Significant (p-value < 0.05), ** significant (p-value < 0.001).

## Discussion

This study was conducted to assess time to treatment failure and its predictors among children receiving first-line ART in Tigray Region public general hospitals. The treatment failure of this study was 13.4% (95% CI, 10.43–17.08), with an incidence rate of 3.3 (95% CI, 2.6–4.3) per 1000 child-month observation or 39.6 per 1000 child-year observation. The median time to treatment failure was greater than or equal to 75 months, with a cumulative failure probability of 35.7% at the end of the study. Poor ART adherence, baseline CD4 count below 200 cells/mm³ and 200–350 cells/mm³, and NVP-based initial regimen were predictors of first-line ART treatment failure.

The incidence rate of first-line ART treatment failure was 3.3 (95% CI, 2.6–4.3) per 1000 child-months of observation. This finding was in line with a study conducted in Addis Ababa Kolfe Keranyo Sub-City (3.45 per 1000 child-months of observation) [13], Oromia (4.2 per 1000 child-months of observation) [26], and Welayta (3.2 per 1000 child-months of observation) [15]. But it is found to be higher than the finding in the Amhara region (2.2 per 1000 person-months of observations) [14]. The possible explanation for the variation in the Amhara Regional State could be the variation in person-time observations. The total person-time observation was 28,562.5 person-months, which is much larger than the 16,532 person-months of observation in the current study. Another explanation for this could be that the failure rate in the Amhara region is solely determined by immunologic and clinical criteria, which could further lengthen the time to detection compared to viral load criteria. Moreover, it is lower than the findings from a study conducted in Tigray (8.7 per 1000 person-months of observation) [20] and Mozambique and Uganda (16.6 per 1000 child-months of observation) [27]. The possible reason for the difference in Tigray might be due to the difference in person-time of observation, duration of follow-up, and study setting (limited study area), which were only conducted in three hospitals in Tigray, which may be underestimated. In Mozambique and Uganda, the difference could be attributed to the era of the study as well as disparities in sociodemographic traits.

The proportion of treatment failures in this study was 13.4% (95% CI, 10.43–17.08). Of these failures, 31 (56.4%) were virological failures, 7 (12.7%) were immunological failures, 5 (9.1%) were clinical, 8 (14.5%) were immunological and virological, 3 (5.4%) were immunological and clinical, and 1 (1.8%) was virological and clinical. The reason for the highest proportion of virological failure might be that virological failure earlier detects treatment failure than immunological and clinical failures [5]. The proportion of treatment failure in this study was similar to a study conducted in Amhara Region Referral Hospitals (12.19%) [16], Gondar Comprehensive Specialized Hospital (14%) [11], North-West Ethiopia (13.5%) [10], Rwanda (16%) [28], and Cameroon (17%) [29]. But higher than a study conducted in the Amhara Regional State (7.7%) [14] and Ghana (6.5%) [30]. The reason for the higher proportion might be explained by the fact that a study conducted in the Amhara regional state used only clinical and immunological criteria to classify treatment failure. In this study, in addition to clinical and immunological criteria, virological failure criteria were also used, which could increase the detection of treatment of failed children. The difference in Ghana may be explained by the sample size and definition of treatment failure. A study conducted in Ghana used a small sample size, and the definition of treatment failure only used virological criteria with strict adherence and consecutively measuring viral load up to eight times.

The proportion of treatment failure in our study was lower than in a study conducted in Togo (67%) [8], Cameron (53%) [31], Addis Ababa (17.2%) [13], and in Mekelle and south Tigray (23.8%) [20]. The difference in Togo (67%) and Cameroon (53%) might be explained by variations in the diagnostic standards for treatment failure as well as disparities in sociodemographic traits. Hence, the above studies were determined using viral load to diagnose treatment failure that was identified earlier than clinical and immunological indicators. In Ethiopia, diagnosis of treatment failure using viral load began recently; it could influence the rate of treatment failure relative to other countries. The difference in Addis Ababa and South Tigray could be explained by selecting study participants, in which some of the study participants stayed pre-ART and might have advanced HIV/AIDS during starting ART, which might increase the occurrences of treatment failure. Whereas in this study, the study participants are selected after a test-and-treat strategy is implemented, which could result in a better outcome and a lower chance of developing treatment failure.

The median time to treatment failure was greater than or equal to 75 months, with a cumulative failure probability of 35.7% at the end of the study. It is higher than the results of studies conducted in Shashemene and Tigray, which found that the median survival times were 30 and 57.7 months, respectively [10,18,20]. This might be explained by the study participants of other studies who included both before and after the test-and-treatment guidelines were implemented. Whereas in this study, the study participants were selected after test-and-treatment guidelines were implemented. That means they started ART irrespective of their baseline CD4 count or WHO stage, which could result in a better outcome and delay the onset of treatment failure. In addition, this could be due to the improvements in therapeutic and diagnostic measures in the current visits from an increase in access and variety of more potent drugs nowadays than in the past periods [5].

In this study, poor adherence among children receiving first-line ART had a 3.6 times higher risk of developing treatment failure compared to good adherence (AHR = 3.6, 95% CI: 1.7–7.5). This finding is supported by studies conducted in Weldia, Amhara referral hospitals, and Addis Abeba [10,13,14]. This could be explained by the fact that the success of ART depends on adherence to the treatment regimen [5]. Poor adherence decreases drug effectiveness, which declines immunity; this will in turn increase the risk of opportunistic infection and drug resistance [32]. This in turn increases rates of viral replication, high rates of CD4 destruction, accumulations of resistant viruses, and faster rates of disease progression. This overall results in treatment failure. But this finding is not consistent with the studies conducted at Tikur Anbessa Specialized Hospital [33] and Shashemene Town Health Facilities [18]. The reason might be due to the better quality of ART services and the improved awareness of patients’ families to comply with the advice of the counselors.

In this study, baseline CD4 counts of <200 cells/mm³ and 201–350 cells/mm³ had 3.8- and 2.7-times higher risk of developing ART treatment failure compared to patients with more than 500 cells/mm³ CD4 counts (AHR = 3.8, 95% CI: 1.8–8.5) and (AHR = 2.7, 95% CI: 1.2–6.2), respectively. This finding is consistent with a study conducted in Oromia [26], which found that children with <200 cells/mm³ and 201–350 cells/mm³ were 2.55 and 2.44 times more likely to develop ART treatment failure, respectively. The current finding is also supported by prior studies conducted in Welayta and Gonder specialized referral hospitals [11,15]. The possible explanation might be that patients with a low or very low CD4 count are more likely to have different opportunistic infections, and the added burden of diseases further complicates their treatment responses. In addition, as CD4 counts decline, viral replication accelerates, resulting in the rapid accumulation of drug resistance. The circulation of such a resistant virus increases the risk of treatment failure [4].

Children treated with an initial NVP-based regimen were 4 times at higher risk of treatment failure compared to those treated with a non-NVP-based regimen (AHR = 4, 95% CI: 1.8–8.8). This finding was supported by studies conducted in Addis Ababa, Shashemene, and South Africa [13,18,19]. This could be explained by the fact that children taking NVP-based regimens are more likely to develop drug resistance, particularly to NVP. This is because NVP has a low genetic barrier to resistance, which can lead to treatment failure [34]. In addition, children may have reduced absorption, increased clearance, or drug-drug interactions that lead to subtherapeutic NVP levels, increasing the risk of treatment failure [35]. However, this finding is contrary to a study conducted in the Amhara Regional State, in southern Tigray, and in the Oromia region [12,14,20]. The finding regarding NVP-based regimens as predictors of treatment failure in first-line ART for children can be attributed to the complex interplay of pharmacokinetic factors, adherence, any resistance they might have before starting treatment, and the availability of other treatment options [34,35].

In this study, covariates that were not predicting first-line ART failure were baseline WHO stage, baseline opportunistic infections, hemoglobin [12], being malnourished [17], caregiver being widowed [15], being orphaned [10,12], and missed CPT [10]. In most studies, first-line ART treatment failure was higher among children with advanced WHO stages III and IV [10,11,31] and baseline opportunistic infections [16,20]. But in this study, those variables were not predictors of treatment failure for first-line ART. This could be because those studies were conducted before the test-and-treat strategy was implemented, which might result in a higher number of children with advanced WHO clinical stages being enrolled in their studies, which could lead to treatment failure. In addition, not taking CPT, being orphaned, being anemic, and having malnutrition were predictors of treatment failure in other studies but not in this study. This inconsistency might be associated with the method of enrollment because, in this study, the enrolled study participants were after a test-and-treat strategy was implemented. In addition, other reasons could be due to the fact that the health care system in Ethiopia has undergone decentralization, task shifting, and delegation of HIV/AIDS services to front- and mid-level health care providers.

Since the design was retrospective and used secondary data, the study did not exhaustively explore all predictor variables that may influence time to treatment failures, such as some laboratory assessments (e.g., viral load).

## Conclusion and recommendations

The incidence of treatment failure among children receiving first-line ART in Tigray region public general hospitals was found to be high according to the UNAIDS virological suppression targets. The median time to treatment failure was greater than or equal to 75 months. Poor adherence, lower baseline CD4 count, and initial NVP-based regimens were significantly associated with time to treatment failure of first-line ART. Therefore, strengthening adherence support systems and closely monitoring children on ART, mainly those who had low CD4 counts and NVP-based regimens when starting ART, are recommended for early detection and management of treatment failure. Moreover, a longitudinal prospective study may be recommended to follow HIV-positive children on ART that would be helpful to identify other possible predictors (e.g., viral load).

## Supporting information

S1 Appendix I Cox-Snell residual Nelson-Alen cumulative hazard graph of children receiving first-line antiretroviral therapy. (PDF)

S2 Appendix II Schoenfeld residual test for Cox proportional hazard model assumption. (PDF)

S3 Checklist Data extraction. (PDF)
