# Supplementary material for: Time to treatment failure and its predictors among children receiving first-line antiretroviral therapy in Tigray Region public general hospitals, North Ethiopia, 2024: Retrospective cohort study
Source: PLoS One. 2026 Jan 12;21(1):e0339269. doi: 10.1371/journal.pone.0339269 (PMC12795365; doi:10.1371/journal.pone.0339269)

Appendix I: Cox-Snell residual Nelson-Alen cumulative hazard graph of children receiving first-line antiretroviral therapy in Tigray region public general hospitals, north Ethiopia, from January 1, 2014, up to March 31, 2020, and from February 1, 2023, to August 31, 2023.

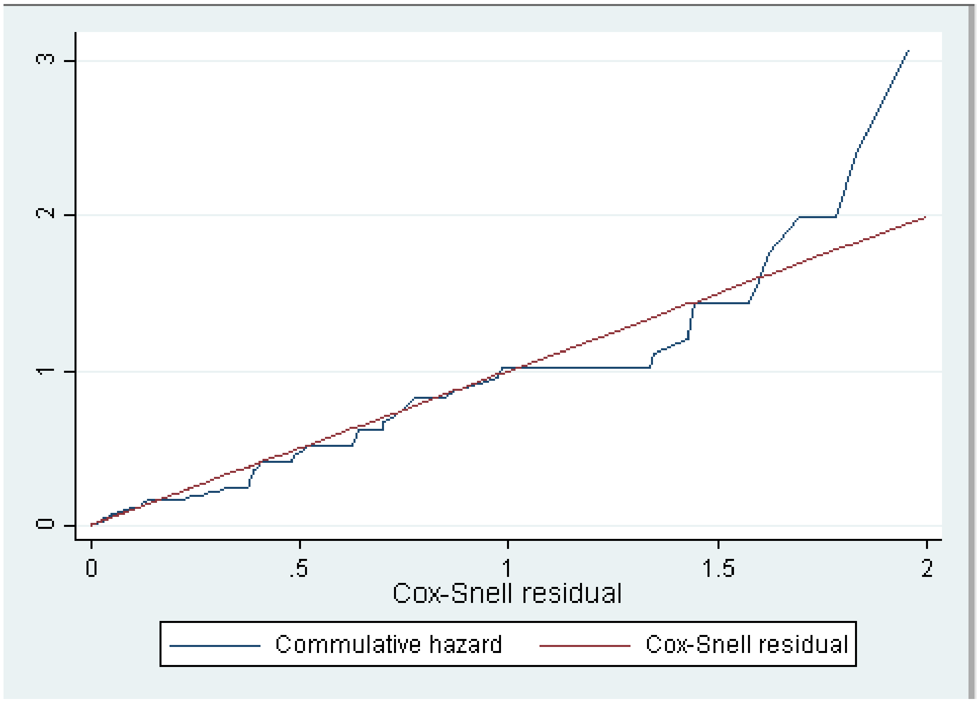

Supplement: S1 Appendix I — (PDF) [file pone.0339269.s001.pdf]
