# Supplementary material for: Time to treatment failure and its predictors among children receiving first-line antiretroviral therapy in Tigray Region public general hospitals, North Ethiopia, 2024: Retrospective cohort study
Source: PLoS One. 2026 Jan 12;21(1):e0339269. doi: 10.1371/journal.pone.0339269 (PMC12795365; doi:10.1371/journal.pone.0339269)
Supplement: S2 Appendix II — (PDF) [file pone.0339269.s002.pdf]

Appendix II: Schoenfeld residual test for Cox proportional hazard model assumption of each covariate and overall model of the Cox proportion hazard.

**Covariates**	**Rho**	**Chi2**	**Df**	**p-value**
Sex	0.07023	0.31	1	0.5795
Marital status	0.20566	2.09	1	0.1478
Opportunistic Infection	-0.05973	0.1	1	0.7497
Caregiver serology status	0.09284	0.43	1	0.5138
Baseline hemoglobin level	0.14316	1.19	1	0.2752
Baseline WHO stage	0.02950	0.03	1	0.8734
Baseline CD4 count	0.05152	0.17	1	0.6777
ART Adherence	0.14517	1.47	1	0.2253
Initial ART regimen	0.00058	0.00	1	0.9965
BAZ	0.11385	0.69	1	0.4066
HFA	0.17556	1.99	1	0.1583
ART side effect	0.03174	0.06	1	0.8114
Global test		9.66	12	0.6458
