# Supplementary material for: Time to treatment failure and its predictors among children receiving first-line antiretroviral therapy in Tigray Region public general hospitals, North Ethiopia, 2024: Retrospective cohort study
Source: PLoS One. 2026 Jan 12;21(1):e0339269. doi: 10.1371/journal.pone.0339269 (PMC12795365; doi:10.1371/journal.pone.0339269)
Supplement: S3 Checklist — (PDF) [file pone.0339269.s003.pdf]

Appendix-I: Data extraction checklist

This format is adapted from related studies and self-developed from reviewing HIV/AIDS treatment guidelines, ART registration booklets, and ART monitoring multi-charts. It is prepared for the collection of sociodemographic characteristics, clinical, laboratory, ART follow-up, and other medication information that are important for the assessment of time to treatment failure and its predictors among children receiving first-line antiretroviral therapy in Tigray region general hospitals, north Ethiopia, 2023. All the information will be retrieved from the clients ART registration by trainee BSc nurses working outside of the selected study hospitals without mentioning the names of the clients.

NB: Please mark the circle on the correct answer from the option numbers.

Part I: **Sociodemographic characteristics**

Code______ No.___________ MRN_______________ Unique ART No._____________ Hospital _________

S.no	Child sociodemographic characteristics	Possible answer	remark
101	sex	1. Male 2. Female	
102	age at ART initiation	_________________________MM/YY	
Care giver sociodemographic characteristics		Possible answer	
103	Residency	1. Urban 2. Rural	
104	Marital status	1. Single 2. Married 3. Divorced 4. Widowed	
105	Level of education	1. No education 2. Primary 3. Secondary 4. Tertiary 5. Others__________	
106	Occupation	1.Farmer 2. Marchant 3. Governmental employee<br>4. Daily laborer 5.House wife 6. Others _______________	
107	Religion	1.Ortodox 2. Muslim 3. Protestant 4.Catholic 5. Other__________	
108	primary caretaker	1. Both parents 2. Mother 3. Father 4. Relatives<br>5. Neighbors 6. Orphanage	

109	care giver serology status	1. Negative 2. Positive 3. Unknown	
Part II: Baseline Clinical, laboratory and nutritional characteristics			
201	WHO clinical stage	1. I 2. II 3. III 4. IV	
202	CD4 count at initiation count/percent	(__________________cells/mm3), _____________%	
203	Viral load	______________________________copies/ml	
204	Baseline Opportunistic infection	1. No 2. Yes, if "No" skip to 206	
205	If yes what opportunistic infection is	1. TB 2. Diarrhea 3. Pneumonia 4. Meningitis<br>5. Skin disorders 6. URTI 7.SAM 8. Others, specify________________________	
206	Hemoglobin level	(___________________mg/dl)	
207	Weight, height	(______________________kg)<br>(___________________cm)	
208	Baseline developmental status < 5 years	1. Appropriate 2. Delayed 3. regression	
209	Baseline functional status for ≥5 years children	1. Working 2. Ambulatory 3. Bedridden.	
ART follow up and other medication characteristics		Possible answer	
301	ART regimen at first	1.D4t-3TC-NVP 2. D4t-3TC-EFV 3. AZT-3TC-EFV 4.AZT-3TC-NVP<br>5. TDF-3TC-EFV 6.AZT-3TC-LPV/R 7. ABC-3TC-LPV/R 8. Others________________	
302	ART adherence at least the last six month)	1. Good 2. Fair 3. poor	
303	disclosure status	1. Yes 2. No	
304	ARV prophylaxis for PMTCT	1. Given 2. Not given	
305	isoniazid prophylaxis	1. Given 2. Not given	

306	cotrimoxazole prophylaxis	1. Given 2. Not given	
307	ART treatment failure (status)	1.yes 2. No, if “No” skip to 309	
308	if yes, types of ART treatment failure	1. Immunological 2. Clinical 3. Virological 4. Immunological and clinical 5. Immunological and virological 6. Clinical and virological 7. Mixed of all	
309	regimen change/substitution	1. yes 2. No, if “No” skip to 311	
310	reason of regimen change/substitution	1. Treatment failure 2. Drug toxicity<br>3. For TB 4. New regimen 5. Others	
311	ART Side effect	1. Yes 2. No, if “No” skip to 313	
312	If yes, type of side effect	1.Nausea 2. Fatigue 3. Headache 4. Rash 5. Anemia 6. Others	
313	Date of ART initiation	(____________________________DD/MM/YY)	
314	time to first line ART failure	(_________________in months)	
315	duration of ART used	(_____________in months)	
316	patient status at last visit	1. Lost to follow up 2. Died 3. transfer out 4. On ART	
